# Diagnostic practice and awareness of SDH- and FH-deficient renal cell carcinoma: results from an Italian Study Group of uropathology (GIUP) survey

**DOI:** 10.1007/s00428-026-04431-3

**Published:** 2026-02-04

**Authors:** Giuseppe Nicolò Fanelli, Anna Caliò, Stefano Marletta, Maria Ballotta, Marco Barella, Guido Bellezza, Paola Bianco, Angelo Giovanni Bonadio, Piergiuseppe Colombo, Alessandro D’Amuri, Giovanni De Chiara, Veronica Errigo, Denise Fiorini, Francesca Franzi, Daniele Liscia, Lisa Marcolini, Daniela Onnis, Francesca Pagliuca, Antonio Paniccià Bonifazi, Francesco Pierconti, Barbara Pozzi, Lavinia Stefanizzi, Marina Valeri, Mariavittoria Vescovo, Guido Martignoni

**Affiliations:** 1 Division of Pathology, Department of Translational Research and New Technologies in Medicine and Surgery, University of Pisa, Pisa, Italy; 2 First Division of Pathology, Pisa University Hospital, Pisa, Italy; 3 Department of Pathology and Laboratory Medicine, Weill Cornell Medicine, New York, NY USA; 4 Department of Diagnostics and Public Health, Section of Pathology, University of Verona, Largo L. Scuro 10, 37134 Verona, Italy; 5 Division of Pathology, Humanitas Istituto Clinico Catanese, Catania, Italy; 6 Department of Biomedical Sciences, Humanitas University, Milan, Italy; 7 Pathology Unit, AULSS 5 Polesana, S. Maria Della Misericordia Hospital, Rovigo, Italy; 8 Pathology Unit, Fondazione IRCCS Istituto Nazionale Dei Tumori, Milan, Italy; 9 Pathology Unit, Department of Medicine and Surgery, University of Perugia, Perugia, Italy; 10 Pathology Unit, Department of Medical Sciences and Public Health, University of Cagliari, Cagliari, Italy; 11 Pathology Unit, S. Giacomo Hospital, Novi Ligure, Italy; 12 Pathology Department, IRCCS Humanitas Research Hospital, Milan, Italy; 13 Pathology Unit, “Vito Fazzi” Hospital, Lecce, Italy; 14 Pathology Unit, Department of Experimental Medicine, University of Salento, Lecce, Italy; 15 Pathology Unit, A.O.R.N. “San Giuseppe Moscati”, Avellino, Italy; 16 Pathology Unit, Department of Pathology, San Paolo Hospital – ASL2 Liguria, Savona, Italy; 17 Pathology Unit, Pederzoli Hospital, Peschiera del Garda, Italy; 18 Pathology and Histology Unit, ASST Sette Laghi, Circolo and Fondazione Macchi Hospital, Varese, Italy; 19 Pathology Unit, Biella Hospital, ASL Biella, Biella, Italy; 20 Pathology Unit, ARNAS “G. Brotzu”, Cagliari, Italy; 21 Pathology Unit, University of Campania “Luigi Vanvitelli”, Naples, Italy; 22 Pathology Unit, ASL Roma 1, Rome, Italy; 23 Institute of Pathology, Fondazione Policlinico Universitario A. Gemelli IRCCS and Catholic University of the Sacred Heart, Rome, Italy; 24 Pathology Unit, ASST Lecco, Lecco, Italy; 25 Pathology Unit, ASST Bergamo Est – Bolognini Hospital, Seriate, Bergamo, Italy; 26 Pathology Unit, Fondazione Policlinico Universitario Campus Bio-Medico, Rome, Italy

**Keywords:** SDH-deficient RCC, FH-deficient RCC, Hereditary renal cancer

## Abstract

Metabolic renal cell carcinomas (RCC) deficient in succinate dehydrogenase (SDH) or fumarate hydratase (FH) are rare but clinically significant entities formalized in the last WHO classification. Their recognition typically starts from morphology and is corroborated by targeted immunohistochemistry (IHC) and, where appropriate, molecular and germline testing. In routine practice, however, implementation may be uneven. Hence, we conducted a nationwide, web-based survey (made by 25 items) among members of the Italian Study Group of Uropathology (GIUP) to map real-world awareness, diagnostic pathways, and test availability across Italian centers. Twenty-one pathologists responded; 18/21 (85.7%) reported dedicated uropathology practice with heterogeneous seniority (≤ 5 years, 28.6%; 5–10 years, 19.0%; 10–20 years, 14.3%; > 20 years, 38.1%). Despite substantial renal-tumor workloads (12/20, 57.1% handled > 100 cases in the previous 5 years), direct exposure to metabolic RCCs remained limited (FH-deficient ≥ 1 case, 11/21, 52.4%; SDH-deficient ≥ 1 case, 9/21, 42.9%). Suspicion was predominantly morphology-led: for SDH-deficient RCC, morphology ranked first in 16/21 (76.2%) with the commonest sequence morphology > age > number of lesions (76.2%); for FH-deficient RCC, morphology was top-ranked in 19/21 (90.5%). Key morphologic cues were mixed architectural patterns/papillary elements/macronucleoli for FH-deficient RCC, and eosinophilic cytoplasm with solid–alveolar architecture for SDH-deficient RCC. IHC mirrored these priorities (FH top for FH-deficient, 85.7%; SDHB top for SDH-deficient, 76.2%), whereas 2-succinocysteine (2SC) was rarely available (1/21, 4.8%). Critically, this FH-loss-only workflow can miss non-truncating FH variants (FH immunoreactive but enzymatically inactive) tumors, contributing to under-recognition. Molecular testing would be requested in all suspected cases by 12/21 (57.1%); among selective users, equivocal IHC was the leading trigger (6/8, 75%). Overall, metabolic RCC recognition in Italy is primarily morphology-driven but constrained by uneven access to confirmatory IHC, particularly 2SC, and to molecular assays. The findings argue for harmonized diagnostic algorithms, regional reference laboratory networks, and routine involvement of molecular tumor boards, supported by targeted educational initiatives (including curated digital slide repositories), to standardize practice and improve patient pathways from morphologic suspicion to genetic counselling and tailored surveillance.

**Supplementary Information:**

The online version contains supplementary material available at https://doi.org/10.1007/s00428-026-04431-3.

## Introduction

The 2022 World Health Organization (WHO) classification of renal tumors refined the framework of “*molecularly defined renal cell carcinomas*” (RCC), which includes entities driven by specific genetic and metabolic alterations rather than solely by morphological features. Among these, succinate dehydrogenase (SDH)-deficient and fumarate hydratase (FH)-deficient RCC have become key examples of metabolic renal tumors [1, 2].

**Fig. 1:**
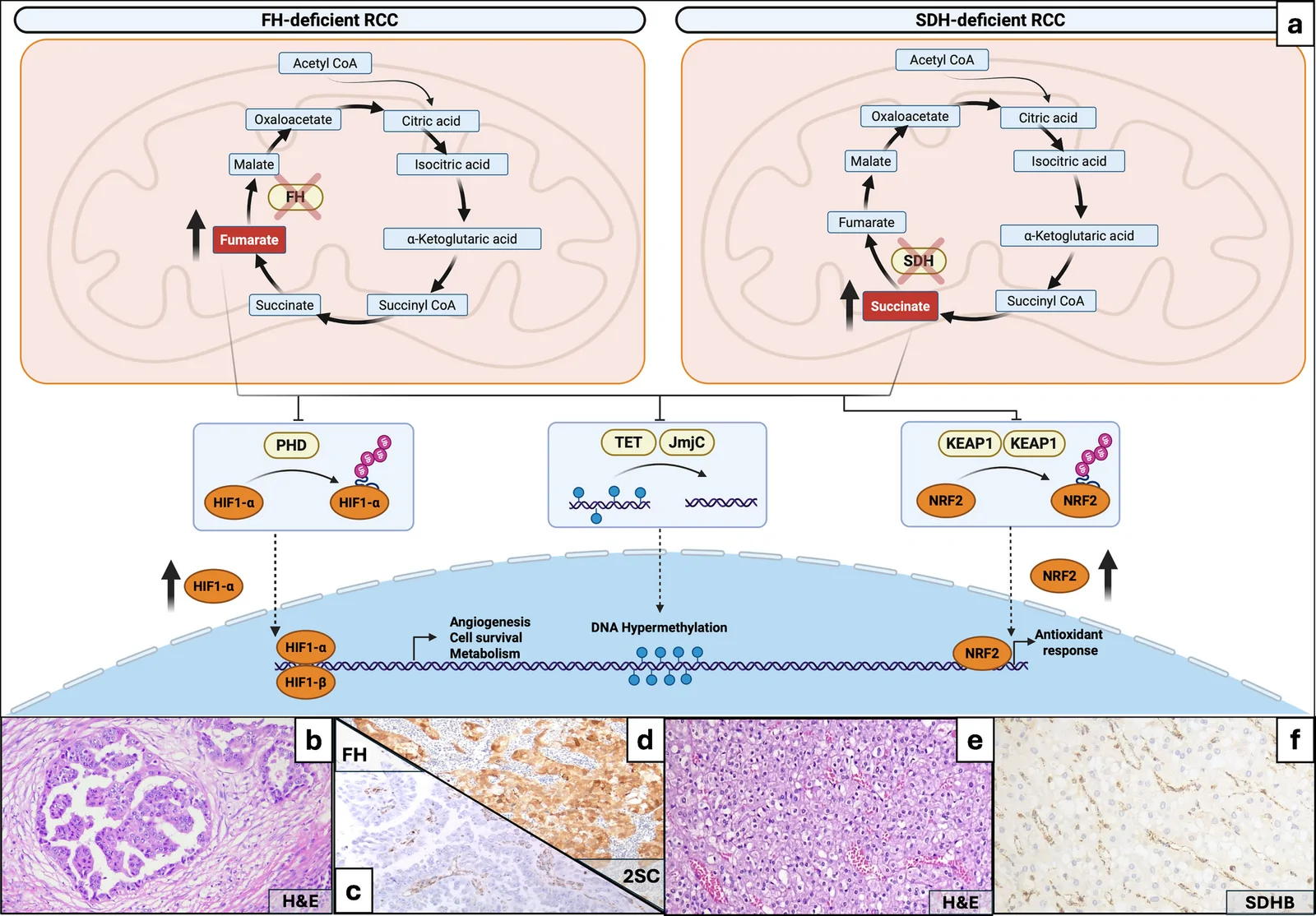
Metabolic and pathological framework of FH-deficient and SDH-deficient renal cell carcinoma. Top: schematic of the TCA cycle highlighting enzymatic loss of FH (left) or SDH/complex II (right) with consequent intracellular accumulation of the oncometabolites fumarate or succinate. Shared downstream effects include inhibition of 2-oxoglutarate–dependent dioxygenases (PHD, TET, JmjC), leading to HIF-α stabilization (pseudohypoxia) and DNA/histone hypermethylation; activation of the NRF2 stress-response pathway via KEAP1 modification is also depicted. Bottom: representative diagnostic workflow elements. In FH-deficient RCC, H&E commonly shows eosinophilic cells with prominent macronucleoli (**b**); IHC demonstrates loss of FH (**c**) and strong 2-SC positivity as a surrogate of fumarate excess (**d**). In SDH-deficient RCC, H&E typically shows solid/nested oncocytic cells with architectural uniformity (**e**); IHC shows loss of SDHB, indicating SDH-complex deficiency regardless of the mutated subunit (**f**). These stains complement routine morphology for case triage and confirmation. RCC, renal cell carcinoma; FH, fumarate hydratase; SDH, succinate dehydrogenase; TCA, tricarboxylic acid cycle; PHD, prolyl hydroxylase; TET, ten-eleven translocation DNA demethylase; JmjC, Jumonji-C histone demethylase; HIF, hypoxia-inducible factor; KEAP1, Kelch-like ECH-associated protein 1; NRF2, nuclear factor erythroid 2-related factor 2; 2-SC, 2-succinocysteine; H&E, hematoxylin and eosin; IHC, immunohistochemistry

SDH-deficient and FH-deficient RCCs collectively account for less than 1% of renal carcinomas and are defined by the inactivation of mitochondrial enzymes that play crucial roles in cellular energy metabolism. SDH is a component of the respiratory chain complex II, linking the tricarboxylic acid cycle with oxidative phosphorylation, while FH catalyzes the reversible hydration of fumarate to malate within the Krebs cycle. Loss of function of either enzyme results in intracellular accumulation of its respective oncometabolite (succinate or fumarate) [3, 4]. These molecules act as competitive inhibitors of 2-oxoglutarate-dependent dioxygenases, including prolyl hydroxylases and histone demethylases, thereby inducing a pseudohypoxic state, stabilizing HIF1α, activating the NRF2 pathway, inducing global DNA and histone hypermethylation, and promoting profound epigenetic reprogramming that promotes tumorigenesis [4] (Fig. 1).

From a clinical standpoint, both entities can occur sporadically or as part of hereditary tumor syndromes. SDH-deficient RCC typically arises in the context of germline mutations in *SDHA*, *SDHB*, *SDHC*, or *SDHD* genes and may coexist with paraganglioma or pheochromocytoma within the spectrum of SDH-related hereditary syndromes [4, 5]. FH-deficient RCC, on the other hand, represents the renal component of hereditary leiomyomatosis and renal cell cancer (HLRCC) syndrome, caused by germline inactivating mutations of the *FH* gene. Cutaneous and uterine leiomyomas and an aggressive form of RCC with early metastatic potential characterize this syndrome [6, 7]. In recent years, sporadic forms of FH-deficient RCC without identifiable germline mutations have also been recognized, further broadening the clinical and pathological spectrum of this entity [7].

Hence, recognition of these neoplasms carries major clinical implications. Accurate identification triggers genetic counseling and germline testing, allows appropriate surveillance of patients and their relatives, and provides prognostic and potentially therapeutic information. FH-deficient RCC, for example, is associated with a poor prognosis and resistance to standard VEGF- or mTOR-directed therapies [8], but may be sensitive to PARP and HIF2α inhibitors [4, 9], mandating prompt recognition and multidisciplinary management. Conversely, SDH-deficient RCC generally follows a more indolent course and may not share these therapeutic vulnerabilities, underscoring a less disrupted redox balance and the biological distinctiveness of these metabolic RCC subtypes; however, it may signal an inherited *SDH* mutation, warranting genetic investigation and family screening [10].

From a diagnostic perspective, these tumors remain challenging in renal pathology. Their morphologic features frequently overlap with those of other eosinophilic or oncocytic renal neoplasms, including oncocytoma, chromophobe RCC, papillary RCC, collecting duct carcinoma, eosinophilic solid and cystic RCC, and unclassified RCC [11]. SDH-deficient RCC often displays a solid, nested, or tubular architecture with bland nuclei and vacuolated cytoplasm, occasionally containing flocculent eosinophilic inclusions [10, 12–14], whereas FH-deficient RCC exhibits large eosinophilic cells with prominent “cherry-red” nucleoli, perinucleolar clearing, and variable papillary, tubulo-cystic, or solid patterns [15–17] (Fig. 1b, e). Necrosis, sarcomatoid differentiation, and prominent mitotic activity are frequent in FH-deficient cases, underscoring their aggressive nature.

Immunohistochemistry (IHC) plays a central role in diagnosis but remains inconsistently available across pathology laboratories. Loss of SDHB staining is diagnostic of SDH deficiency and implies a defect in any subunit of the SDH complex (Fig. 1f). Similarly, loss of FH expression indicates FH deficiency, and positivity for 2-succinocysteine (2SC) serves as an indirect marker of fumarate accumulation [3, 7, 18, 19] (Fig. 1c, d). However, due to the limited availability of validated antibodies (Abs) and interlaboratory differences in technical performance, these stains are not routinely included in the diagnostic work-up of renal eosinophilic tumors in many institutions. Moreover, confirmatory molecular testing for *SDHx* or *FH* gene alterations is often confined to specialized centers, leading to underdiagnosis of these rare entities.

The rarity of SDH- and FH-deficient RCC translates into limited practical exposure for most pathologists, resulting in heterogeneous awareness and variable confidence in recognizing their morphologic and immunophenotypic clues. In addition, the integration of hereditary counseling into diagnostic workflows remains inconsistent, often depending on local expertise and institutional policies. Consequently, the true incidence and recognition rate of these metabolic RCCs are likely underestimated in routine practice.

Although the scientific literature on SDH- and FH-deficient RCC has expanded considerably over the past decade, most publications consist of small case series or single-institution studies, and information on real-life diagnostic practice is scarce. Data regarding how often pathologists suspect these entities, which IHC markers are available, and how results are interpreted remain largely anecdotal. This lack of structured knowledge represents a practical gap between theoretical understanding and diagnostic implementation. To address this unmet need, the Italian Study Group of Uropathology (GIUP), operating under the auspices of the Italian Society of Anatomic Pathology and Cytology (SIAPeC-IAP), conducted a nationwide survey in 2024 aimed at capturing the current Italian experience with SDH- and FH-deficient RCC, as previously done for other rare entities [20]. The questionnaire explored diagnostic awareness, morphological and immunohistochemical criteria used to raise suspicion, and the availability of molecular testing and genetic referral pathways. By mapping the real-world scenario across academic and hospital laboratories, the study sought to identify common diagnostic patterns, technical limitations, and educational needs, ultimately providing a foundation for future harmonization of diagnostic practice and quality standards in the assessment of these rare metabolic renal tumors.

## Materials and methods

### Survey design

This cross-sectional survey aimed to assess current levels of awareness, diagnostic approach, and technical resources for SDH-deficient and FH-deficient RCC across Italian pathology centers. The survey was created using Google Forms and distributed online to GIUP members between the 21 st of May and the 17th of June 2024. It comprised 25 questions organized into four thematic sections: professional background, diagnostic approach and clinicopathological aspects, immunohistochemistry, and molecular testing.

Five main types of questions were used:

1. Dichotomous closed-ended (Yes/No) to assess the presence or absence of diagnostic practices or findings (e.g., use of 2SC antibody, observation of sarcomatoid dedifferentiation, or request for molecular testing).
2. Single-choice closed-ended questions with predefined categorical options to collect demographic and quantitative information (e.g., years of practice, case volume, number of SDH- or FH-deficient RCCs diagnosed).
3. Ranking (ordinal-scale) questions requiring respondents to order predefined items by diagnostic relevance (e.g., morphology, age, number of lesions, or immunohistochemical markers such as FH, CK7, and AMACR).
4. Multiple response (multi-choice) questions allow more than one valid answer (e.g., diagnostic methods used: morphology, IHC, molecular analysis).
5. Open-ended items, including numerical entries for counts (e.g., number of cases observed) and free-text responses for qualitative comments (e.g., metastatic sites or reasons for selective molecular testing).

The full list of questions is provided in Table 1.

**Table 1:** Overview of the 25-item, web-based questionnaire. For each item, the table lists the domain, exact question, response format, and the options shown to respondents. RCC, renal cell carcinoma; SDH, succinate dehydrogenase; FH, fumarate hydratase; IHC, immunohistochemistry; 2SC, 2-succinocysteine

Query	Type of question	Options
***Professional background***		
How many years of diagnostic practice do you have?	Single-choice closed-ended	< 5; 5–10; 10–20; > 20
Are you specifically dedicated to uropathology in your institution?	Dichotomous closed-ended	Yes; No
How many renal tumour cases have you evaluated in the last five years?	Single-choice closed-ended	< 25; 25–50; 50–100; > 100
How many FH-deficient RCCs have you observed in the last five years?	Single-choice closed-ended	0; < 5; 5–10; > 10
How many SDH-deficient RCCs have you observed in the last five years?	Single-choice closed-ended	0; 1; 2; ≥ 3
***Diagnostic Approach and Clinicopathological Aspects***		
In what order do you rank the following parameters when suspecting SDH-deficient RCC?	Ranking (ordinal-scale)	Age; Morphology; Number of lesions
In what order do you rank the following parameters when suspecting FH-deficient RCC?	Ranking (ordinal-scale)	Age; Morphology; Number of lesions
How do you usually reach a diagnosis of SDH- or FH-deficient RCC?	Multiple response (multi-choice)	Morphology; Immunohistochemistry; Molecular analysis
Which morphological features make you suspect FH-deficient RCC?	Ranking (ordinal-scale)	Mixed architectural patterns; Papillary architecture; Macronucleoli; Eosinophilic cytoplasm
Have you ever observed sarcomatoid dedifferentiation in FH-deficient RCC?	Dichotomous closed-ended	Yes; No
If yes, how many cases?	Open-ended numerical	
How many low-grade FH-deficient RCCs have you diagnosed?	Open-ended numerical	
Among the FH-deficient RCCs you have observed, how many presented with metastases?	Dichotomous closed-ended	Yes; No
If yes, specify the sites	Open-ended text	
Have you ever observed sarcomatoid dedifferentiation in SDH-deficient RCC?	Dichotomous closed-ended	Yes; No
If yes, how many cases?	Open-ended numerical	
Which morphological features make you suspect SDH-deficient RCC?	Ranking (ordinal-scale)	Solid-alveolar architecture; Eosinophilic cytoplasm; Architectural uniformity
How many times have you observed eosinophilic flocculent cytoplasmic inclusions in SDH-deficient RCC?	Single-choice closed-ended	0; 1; > 1
How many times have you observed necrosis in SDH-deficient RCC?	Single-choice closed-ended	0; 1; > 1
How many times have you observed high nucleolar grade (3–4) in SDH-deficient RCC?	Single-choice closed-ended	0; 1; > 1
***Immunohistochemistry***		
In what order do you rank the following IHC markers for FH-deficient RCC?	Ranking (ordinal-scale)	FH; CK7; AMACR
Have you ever used the 2-succinocysteine (2SC) antibody?	Dichotomous closed-ended	Yes; No
In what order do you rank the following IHC markers for SDH-deficient RCC?	Ranking (ordinal-scale)	SDHB; panCK; CK7; CD117
***Molecular testing***		
If molecular testing platforms were available, would you request testing in all suspected cases?	Dichotomous closed-ended	Yes; No
If “No”, under which circumstances would you request it and why?	Open-ended text	

As this study involved anonymized professional data only, it was exempt from ethical approval requirements according to national regulations and the Declaration of Helsinki (2013 revision).

### Data analysis

Responses were exported for descriptive statistical analysis. Frequencies and percentages were calculated for categorical variables, while ranking data were analyzed by identifying the most frequent first-position items and complete ranking sequences. Missing data were excluded from percentage denominators. Plots and graphs were generated with GraphPad Prism (v10.2.0). Images have been created with BioRender.

## Results

Results are summarized in Table 2 and organized by domain.

**Table 2:** Responses from twenty-one participants. For each item, valid respondents are indicated; percentages exclude missing data. “Ranking sequences” report the most frequent complete orders (not all permutations). RCC, renal cell carcinoma; SDH, succinate dehydrogenase; FH, fumarate hydratase; IHC, immunohistochemistry; 2SC, 2-succinocysteine

Domain	Question	Valid response	Response	N	%
Professional background	*Are you specifically dedicated to uropathology in your institution?*	21	Yes	18	85.7
			No	3	14.3
	*How many years of diagnostic practice do you have?*	21	< 5	6	28.6
			5–10	4	19.0
			10–20	3	14.3
			> 20	8	38.1
	*How many renal tumour cases have you evaluated in the last five years?*	20	< 25	1	4.8
			25–50	3	14.3
			50–100	4	19.0
			> 100	12	57.1
	*How many FH-deficient RCCs have you observed in the last five years?*	21	0	10	47.6
			< 5	8	38.1
			5–10	2	9.5
			> 10	1	4.8
	*How many SDH-deficient RCCs have you observed in the last five years?*	21	0	12	57.1
			1	8	38.1
			2	1	4.8
			≥ 3	0	0
Diagnostic approach	*In what order do you rank the following parameters when suspecting SDH-deficient RCC?*	21	***Top-ranked parameter***		
			Morphology	16	76.2
			Age	5	23.8
			Number of lesions	0	0
			***Ranking sequences***		
			Morphology > Age > Number of lesions	16	76.2
			Age > Morphology > Number of lesions	3	14.3
			Age > Number of lesions > Morphology	2	9.5
	*In what order do you rank the following parameters when suspecting FH-deficient RCC?*	21	***Top-ranked parameter***		
			Morphology	19	90.5
			Age	2	9.5
			Number of lesions	0	0
			***Ranking sequences***		
			Morphology > Age > Number of lesions	19	90.5
			Age > Mophology > Number of lesions	2	9.5
	*How do you usually reach a diagnosis of SDH- or FH-deficient RCC?*	21	Morphology	1	4.8
			Morphology + IHC	10	47.6
			Morphology + IHC + Molecular testing	10	47.6
Morphology FH-Deficient RCC	*Which morphological features make you suspect FH-deficient RCC?*	21	***Top-ranked parameter***		
			Mixed architectural patterns	13	61.9
			Papillary architecture	5	23.8
			Macronucleoli	3	14.3
			Eosinophilic cytoplasm	0	0
			***Ranking sequences***		
			Mixed architectural patterns > Papillary architecture > Macronucleoli > Eosinophilic cytoplasm	13	62
			Macronucleoli > Papillary architecture > Mixed architectural patterns > Eosinophilic cytoplasm	4	19
			Papillary architecture > Mixed architectural patterns > Macronucleoli > Eosinophilic cytoplasm	4	19
	*Have you ever observed sarcomatoid dedifferentiation in FH-deficient RCC?*	20	Yes	2	10
			No	18	90
	*If yes how many times?*	2	2	1	50
			3	1	50
	*How many low-grade FH-deficient RCCs have you diagnosed?*	20	Yes	2	10
			No	18	90
	*If yes how many times?*	3	1	3	100
Clinical aspects FH-Deficient RCC	*Among the FH-deficient RCCs you have observed, how many presented with metastases?*	12	Yes	4	33.3
			No	8	66.7
	*If yes how many times?*	4	1	2	50
			3	1	25
			7	1	25
	*Most common sites*		Lymphnode (2), Lung (1), Peritoneum (1), Liver (2), Bone (1)	-	-
Morphology SDH-Deficient RCC	*Which morphological features make you suspect SDH-deficient RCC?*	21	***Top-ranked parameter***		
			Eosinophilic cytoplasm	12	57.1
			Solid-alveolar architecture	9	38.1
			Architectural uniformity	1	4.8
			***Ranking sequences***		
			Eosinophilic cytoplasm > Architectural uniformity > Solid-alveolar architecture	8	38.1
			Eosinophilic cytoplasm > Solid-alveolar > Architectural uniformity architecture	7	33.3
			Solid-alveolar > Eosinophilic cytoplasm > Architectural uniformity architecture	5	23.8
			Architectural uniformity > Eosinophilic cytoplasm > Solid-alveolar architecture	1	1.21
	*Have you ever observed eosinophilic flocculent cytoplasmic inclusions in SDH-deficient RCC?*	19	Yes	5	26.3
			No	14	73.7
	*Have you ever observed necrosis in SDH-deficient RCC?*	19	Yes	1	5.3
			No	18	94.7
	*Have you ever observed high nucleolar grade (3–4) in SDH-deficient RCC?*	19	Yes	1	5.3
			No	18	94.7
	*Have you ever observed sarcomatoid dedifferentiation in SDH-deficient RCC? If yes how many times?*	20	Yes	0	0
			No	20	100
IHC FH-Deficient RCC	*In what order do you rank the following IHC markers for FH-deficient RCC?*	21	***Top-ranked Ab***		
			FH	18	85.7
			CK7	3	14.3
			AMACR	0	0
			***Ranking sequences***		
			FH > CK7 > AMACR	15	71.4
			CK7 > FH > AMACR	3	14.3
			FH > AMACR > CK7	3	14.3
IHC SDH-Deficient RCC	*In what order do you rank the following IHC markers for SDH-deficient RCC?*	21	***Top-ranked Ab***		
			SDHB	16	76.2
			panCK	5	23.8
			CK7	0	0
			CD117	0	0
			***Ranking sequences***		
			SDHB > CK7 > CD117 > panCK	10	47.6
			SDHB > panCK > CK7 > CD117	6	28.6
			panCK > SDHB > CD117 > CK7	3	14.3
			panCK > SDHB > CK7 > CD117	2	9.5
	*Have you ever used the 2-succinocysteine (2SC) antibody?*	21	Yes	1	4.8
			No	20	95.2
Molecular testing	*If molecular testing platforms were available, would you request testing in all suspected cases?*	21	Yes	12	57.1
			No	9	42.9
	*If “No”, under which circumstances would you request it and why?*	8	Equivocal IHC	6	75
			Morphologic and IHC suspicious	1	12.5
			Lack of in house IHC Abs/platforms	1	12.5

### Professional background

**Fig. 2:**
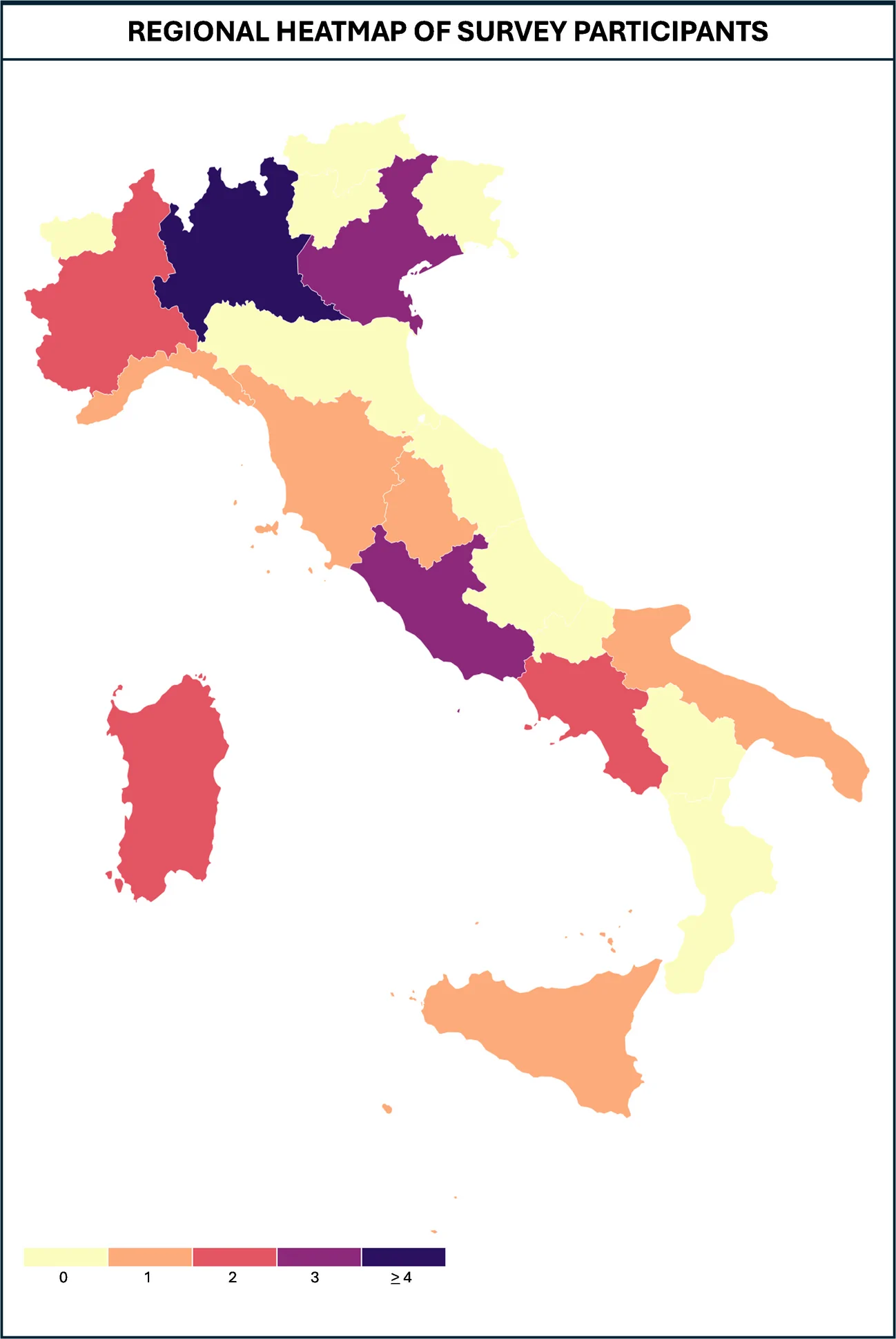
Regional distribution of survey participants. Choropleth map of Italy showing the number of respondents per region. Color intensity reflects respondent count according to the legend (0, 1, 2, 3, ≥ 4). Counts are aggregated by administrative region and sum to the total sample (N = 21). Regions with no respondents are shown in the lightest shade. Islands are included

**Fig. 3:**
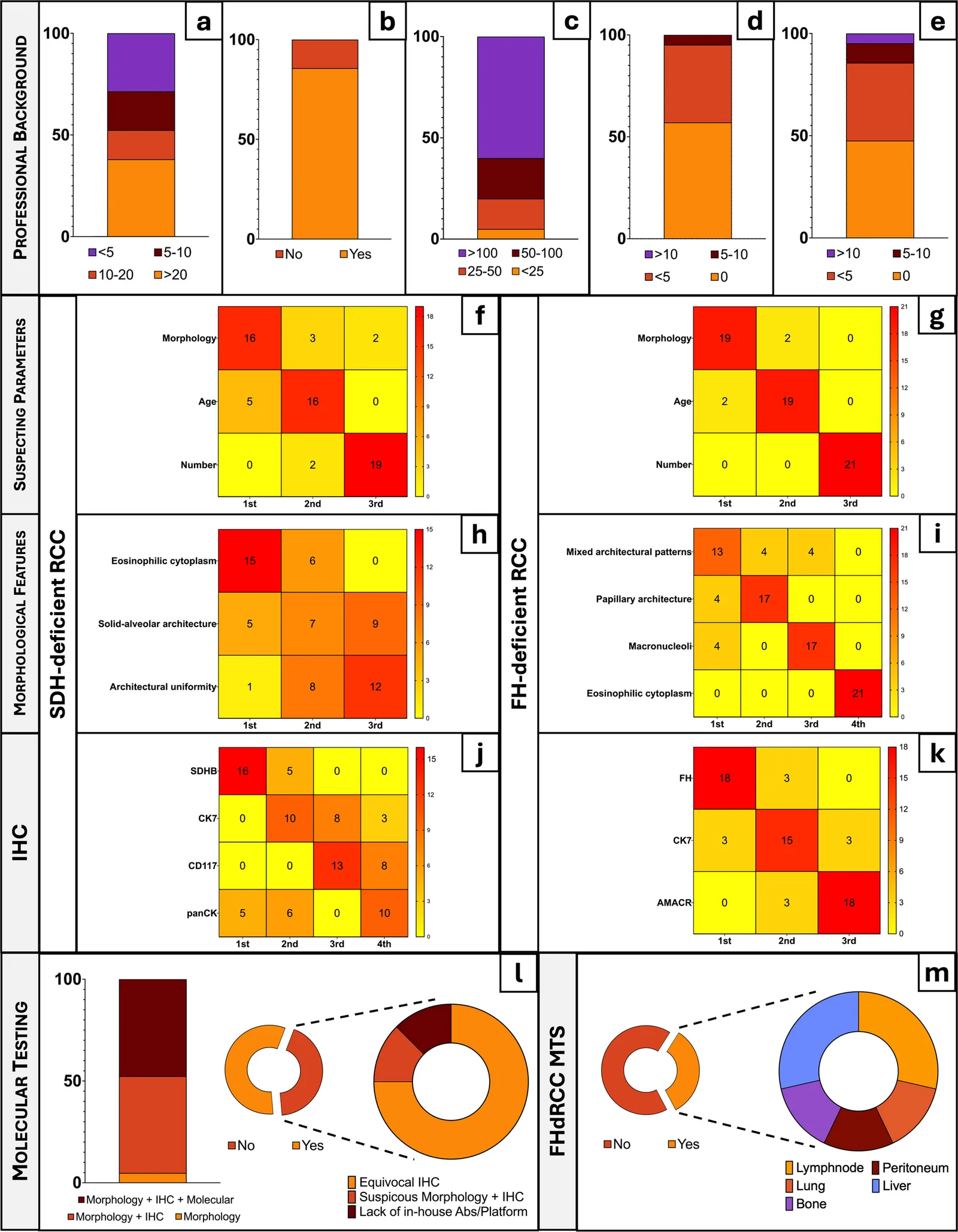
Integrated summary of the survey answers. This composite figure summarizes, at a glance, who responded, how suspicion is raised, which morphologic and immunohistochemistry clues are prioritized, and how molecular testing is used in suspected FH- and SDH-deficient RCC. Panels a–e (Professional background). Stacked bar plots profile respondents by dedication to uropathology (**a**), years in practice (**b**), renal tumor volume in the last 5 years (**c**), and self-reported exposure to FH-deficient (**d**) and SDH-deficient RCC (**e**). Panels f–g (How suspicion is raised). Heatmaps show the rank order (columns =  1 st, 2nd, 3rd) assigned to clinical/pathologic parameters when suspecting SDH-deficient (**f**) or FH-deficient (**g**) RCC (Morphology, Age, Number of lesions). Columns denote rank positions and shading denotes frequency. Panels h–i (Morphological features). Heatmaps summarize the prioritization of histologic clues for SDH-deficient RCC (**h**: solid–alveolar architecture, eosinophilic cytoplasm, architectural uniformity) and FH-deficient RCC (**i**: mixed architectural patterns, papillary architecture, macronucleoli, eosinophilic cytoplasm). Columns denote rank positions, and shading denotes frequency. Panels j–k (Immunohistochemistry). Heatmaps report the diagnostic ranking of antibodies for FH-deficient RCC (**j**: FH, CK7, AMACR) and SDH-deficient RCC (**k**: SDHB, panCK, CK7, CD117). Columns indicate rank position, and shading indicates response frequency. Panel l (Molecular testing). Bar plot shows the intent to request molecular analysis in all suspected cases. For respondents who answered “No (just in selected cases)”, the donut chart summarizes the stated reasons. Panel M (Metastatic presentation in FH-deficient RCC). Of the 12 respondents who provided data, 4 (33.3%) reported metastatic presentation. The right donut chart displays reported sites (mentions, not unique patients): lymph nodes (n = 2), liver (n = 2), lung (n = 1), peritoneum (n = 1), and bone (n = 1). Notes: percentages are calculated on non-missing responses for each item; ranking tasks permitted a single rank position per feature/parameter. These data are descriptive and should be interpreted with caution, given small denominators and potential recall bias. See Table 2 for the complete list of questions and counts

Twenty-one Italian pathologists from 11 regions participated in the 2024 GIUP national survey (Fig. 2). Most respondents (18/21; 85.7%) were actively dedicated to uropathology within their institutions. The majority had substantial diagnostic experience, with eight (38.1%) reporting more than 20 years of practice and only six (28.6%) less than 5 years (Fig. 3a, b). Regarding workload, 12 of 20 valid respondents (57.1%) had evaluated more than 100 renal tumors in the previous 5 years (Fig. 3c). However, the experience with metabolic RCCs was limited: 11 of 21 (52.4%) reported having diagnosed at least one FH-deficient RCC in the last five years, and 9 (42.9%) at least one SDH-deficient RCC (Fig. 3d, e). Only a single respondent had observed more than ten FH-deficient cases.

### Diagnostic approach

When ranking the parameters used to suspect SDH-deficient RCC, morphology was placed first by 16 of 21 respondents (76.2%), followed by age in 5 (23.8%). The most frequent ranking sequence was morphology > age > number of lesions (76.2%), followed by age > morphology > number of lesions (14.3%) (Fig. 3f).

For FH-deficient RCC, morphology was considered the primary clue by 19 of 21 (90.5%), and the combination ranking of morphology > age > number of lesions was almost universal (90.5%) (Fig. 3g).

In terms of workflow, nearly all respondents integrated morphology with ancillary techniques: only one (4.8%) relied on morphology alone, ten (47.6%) combined morphology and IHC, and another ten (47.6%) included molecular testing in selected cases.

### Morphological assessment

For SDH-deficient RCC, eosinophilic cytoplasm was most often ranked first (12/21; 57.1%), followed by solid-alveolar architecture (9/21; 38.1%) and architectural uniformity (1/21; 4.8%) (Fig. 3h). Eosinophilic flocculent cytoplasmic inclusions had been observed by 5 of 19 valid respondents (26.3%), while necrosis and high nucleolar grade (3–4) were each reported by only 1 (5.3%). None had ever observed sarcomatoid dedifferentiation.

For FH-deficient RCC, the most frequently top-ranked feature was mixed architectural patterns (13/21; 61.9%), followed by papillary architecture (5/21; 23.8%) and prominent macronucleoli (3/21; 14.3%) (Fig. 3i). Sarcomatoid dedifferentiation was encountered in 2 of 20 respondents (10%), each reporting 2 or 3 cases, while 2 (10%) had diagnosed low-grade FH-deficient RCCs. Among 12 pathologists who answered questions on clinical behavior, 4 (33.3%) had seen FH-deficient RCCs presenting with metastases, most commonly to lymph nodes (n = 2), liver (n = 2), lung (n = 1), peritoneum (n = 1), and bone (n = 1) (Fig. 3m).

### Immunohistochemistry

For SDH-deficient RCC, SDHB was ranked first by 16 of 21 respondents (76.2%) and pan-cytokeratin by 5 (23.8%). The most common ranking patterns were SDHB > CK7 > CD117 > panCK (47.6%) and SDHB > panCK > CK7 > CD117 (28.6%). Only one pathologist (4.8%) had ever used the 2SC antibody (Fig. 3j).

For FH-deficient RCC, the antibody most frequently ranked first was FH (18/21; 85.7%), followed by CK7 (3/21; 14.3%). The predominant ranking sequence was FH > CK7 > AMACR (15/21; 71.4%) (Fig. 3k).

### Molecular testing

Twelve of 21 respondents (57.1%) indicated that they would request molecular analysis in all suspected SDH- or FH-deficient RCCs, while 9 (42.9%) would restrict testing to selected situations. Among the latter, the most common justification was equivocal immunohistochemistry (6/8; 75%), followed by strong morphological and IHC suspicion (1/8; 12.5%) and lack of in-house immunostaining platforms (1/8; 12.5%) (Fig. 3l).

## Discussion

This nationwide survey provides the first structured assessment of how SDH- and FH-deficient RCCs are recognized, investigated, and reported across Italian pathology laboratories. These tumor types represent paradigmatic examples of metabolic RCCs, initially delineated as separate entities in the 2016 “*WHO Classification of Tumours of the Urinary System,*” and subsequently consolidated in the 2022 WHO update, which placed them within the group of molecularly defined renal neoplasms [1]. Despite this formal nosological recognition, their integration into routine diagnostic workflows remains inconsistent. While awareness of these metabolic entities is widespread among practicing pathologists, practical familiarity and access to confirmatory techniques, particularly specific IHC markers and molecular assays, are uneven across institutions. The survey reveals a paradox typical of rare tumor entities: a high level of theoretical knowledge coexists with limited direct diagnostic exposure. Although most respondents were senior pathologists with longstanding experience in uropathology, more than half had never personally signed out a confirmed FH- or SDH-deficient RCC in the last 5 years. This gap reflects not only the intrinsic rarity of these tumors but also the subtlety of their morphologic spectrum and the reliance on ancillary tools for their recognition. Comparable observations have emerged from recent multi-institutional audits in other countries, which reported under-recognition of metabolic RCCs even within tertiary reference centers and academic consortia [12, 14, 15]. Together, these data underscore the diagnostic challenge posed by metabolic renal neoplasms and justify coordinated efforts, such as the present GIUP initiative, to map national expertise and technical capacities.

The morphological spectrum of SDH- and FH-deficient RCCs represents one of the most intriguing and diagnostically demanding areas in renal tumor pathology; nevertheless, the survey revealed that the morphological features most frequently recognized by participants align with those described in seminal series. For FH-deficient RCC, respondents most often cited mixed architectural patterns, papillary or tubulo-papillary structures, and prominent macronucleoli as key features, corresponding to the well-characterized “cherry-red nucleoli” of RCC in HLRCC syndrome [21]. Such tumors typically display large eosinophilic cytoplasm, frequent necrosis, and a striking combination of nuclear pleomorphism and high mitotic activity. Importantly, only a few respondents reported having encountered putative low-grade FH-deficient RCC, in agreement with current evidence that the majority of these tumors present as high-grade, biologically aggressive lesions, often with early nodal or distant metastases [22]. In contrast, SDH-deficient RCC was generally recognized by its solid–alveolar or nested growth, diffuse eosinophilic cytoplasm, and architectural uniformity, consistent with the prototype delineated by Gill et al*. *[10] and subsequently expanded by Fuchs et al*. *[12]. These tumors often exhibit finely vacuolated cytoplasm and flocculent eosinophilic inclusions, the ultrastructural correlate of abnormal mitochondrial accumulation. However, such inclusions were seldom observed in this survey, most likely reflecting the limited number of confirmed SDH-deficient RCCs encountered, rather than a true absence of the feature, underscoring the experience-dependence of morphologic recognition. These findings illustrate a broader interpretative challenge: morphology alone is rarely sufficient to establish a diagnosis of metabolic RCC. In routine settings, therefore, tumors with incomplete morphologic profiles and without confirmatory IHC or molecular data may prompt many pathologists to default to broader categories, such as unclassified RCC or other oncocytic neoplasms. This tendency likely contributes to the underestimation of SDH- and FH-deficient RCC incidence in both institutional and national datasets. Moreover, the morphologic spectrum of these tumors continues to expand: FH-deficient RCC has been reported in cystic, biphasic, and even deceptively low-grade oncocytic variants, which may lack overt nucleolar prominence or necrosis [21, 22]; similarly, SDH-deficient RCC may display hybrid chromophobe-like or oncocytoma-like areas, creating potential diagnostic pitfalls [12]. This phenotypic plasticity reinforces the need for contextual morphological interpretation, integrating patient age, tumor multifocality, and clinical background with subtle histologic clues.

Furthermore, the survey documents marked inter-laboratory heterogeneity in the diagnostic work-up of metabolic RCCs, particularly in the composition, availability, and interpretation of IHC panels. Although most participants reported access to Abs against FH and SDHB, their routine inclusion in baseline IHC panels for eosinophilic/oncocytic renal neoplasms remains inconsistent. This mirrors the lack of nationally standardized protocols for metabolic RCC testing [17]. Interpretative variability further contributes to diagnostic heterogeneity. Loss of FH or SDHB staining is diagnostically conclusive only when accompanied by appropriate internal positive controls (e.g. vascular endothelium, non-neoplastic tubules, or interstitial cells); in their absence, suboptimal fixation, edge effects, or patchy immunoreactivity can mimic loss and lead to misinterpretation (Supplementary Fig. 1). These pre-analytical and interpretative pitfalls plausibly contribute to under-recognition of metabolic RCCs. Similar observations were made in international series, where a substantial proportion of morphologically suspicious cases lacked confirmatory IHC or molecular documentation of metabolic enzyme loss [12]. This shortfall is consistent with the limited availability of the most sensitive surrogate assay of fumarate accumulation, 2SC IHC, which is rarely implemented outside reference centers; as a result, many laboratories rely on FH loss alone, yielding false-negative results in tumors with missense *FH* variants that retain immunoreactive but inactive protein, further perpetuating under-recognition [3, 15, 19, 23]. Beyond these core metabolic markers, ancillary Abs such as CK7, panCK, CD117, and AMACR remain valuable for establishing the differential diagnosis. FH-deficient RCC is mostly negative for CK7 and variably expresses AMACR [6, 16]. SDH-deficient RCC is usually negative for CK7 and CD117 but can retain epithelial cytokeratin staining, which can be patchy rather than diffuse [12, 14]. Although nonspecific, these conventional Abs, together with emerging biomarkers [24, 25], remain indispensable for defining the broader differential context within which FH- and SDH-deficient RCCs are suspected.

In this context, morphological assessment combined with immunohistochemical profiling remains essential for distinguishing these neoplasms from their principal differential diagnoses. FH-deficient RCC shows broad architectural heterogeneity and can closely mimic several other RCC subtypes. Notably, a substantial proportion of tumors historically signed out as “type 2 papillary RCC” have been reclassified as FH-deficient RCC [26]. Their morphological variability and frequent papillary architecture may also prompt consideration of TFE3/TFEB-altered RCC; however, these latter, in contrast with FH-deficient RCC, frequently express cathepsin K and melanocytic markers (HMB45, MART-1) [27]. FH-deficient RCC may also mimic tubulocystic RCC, once hypothesized to be related [28], but larger size and a mixture of growth patterns typically aid distinction. In all these scenarios, beyond the shared immunohistochemical markers, FH and, when needed, 2SC provide decisive support. SDH-deficient RCC most often enters into differential diagnosis with low-grade oncocytic neoplasms, including oncocytoma, chromophobe RCC, low-grade oncocytic tumor (LOT), hybrid oncocytic tumors, and SDH-like FH-deficient RCC [11]. Hallmark loss of SDHB is the key discriminator; however, ancillary markers help frame the differential diagnoses, such as cytokeratin cocktails expression (e.g., CK8/18, CAM 5.2, CKAE1/AE3), which are often negative or focal [14].

Access to molecular confirmation, via targeted sequencing, NGS panels, or copy-number analysis, remains limited to a minority of Italian centers and typically relies on external referral. In this survey, roughly half of the respondents would request molecular testing in all suspected cases, whereas the remainder would reserve it for equivocal or diagnostically challenging tumors. This selective use reflects infrastructural disparities and the absence of a coordinated national referral pathway for rare renal neoplasms. It also indicates that many laboratories are not yet positioned to adopt emerging technologies such as liquid biopsy workflows, reflex metabolic-RCC panels, or AI-assisted digital pathology pre-screening [29–33].

Taken together, these gaps call for a coordinated service model such as a regional/national hub-and-spoke framework for metabolic RCC. In such a setting, spoke centers could focus on standardizing pre-analytical phases, performing morphology-led triage, and applying a minimal eosinophilic/oncocytic RCC panel under shared standard operating procedures (SOPs). When predefined referral criteria are met (highly suggestive morphology, equivocal or discordant IHC, young age, bilaterality or multifocality, or a suspicious personal or family history), cases could be referred to regional or national hubs, which might concentrate validated metabolic IHC, more specialized assays (such as 2-SC), and integrated genetic evaluation (somatic NGS and, when appropriate, germline SDHx/FH testing with counselling), and could also coordinate multidisciplinary molecular tumor boards.

Such a model could be supported by specimen logistics agreements (SLAs), centralized procurement of niche antibodies, shared validation and external quality assessment (EQA) schemes, digital teleconsultation tools, and a federated registry. Overall, this tiered structure could help to minimize costs and variability, ensure equitable access, and better align real-world workflows with molecular RCC categories, while also facilitating downstream genetic management (timely counselling, cascade testing, and risk-adapted surveillance).

Crucially, these constraints are not merely operational: accurate identification of SDH- and FH-deficient RCCs carries critical implications that extend beyond tumor classification, as these tumors are sentinel lesions for hereditary cancer syndromes, and recognition should trigger genetic counselling, cascade testing, and tailored surveillance for patients and relatives [34]. However, currently, there is no universally accepted threshold for referral to genetic counselling in metabolic RCC. Most guidelines and expert groups recommend germline testing for all patients with confirmed FH-deficient RCC, given the high penetrance of HLRCC syndrome [34]. For SDH-deficient RCC, germline *SDHx* testing and genetic counselling are likewise advised; at minimum, referral should be pursued for patients aged ≤ 46 years, those with bilateral/multifocal tumors, or those with a personal or family history of paraganglioma/pheochromocytoma [35]. In the absence of harmonized criteria, follow-up strategies vary, and cascade testing of at-risk relatives is often incomplete, limitations reflected in the present national survey, where referral to clinical genetics remains inconsistent, frequently depending on local expertise rather than structured protocols. Strengthening these interfaces, potentially through regional molecular tumor boards, is essential to translate morphological recognition into integrated patient care. These findings collectively underscore the need for shared diagnostic guidelines and the establishment of reference laboratories capable of standardizing Abs validation, assay performance, and confirmatory molecular testing within certified quality assurance systems.

Beyond germline risk and prognostic implications, therapeutic considerations underscore the importance of accurate metabolic RCC subtyping. From a surgical-management perspective, hereditary RCC syndromes follow distinct paradigms. For von Hippel–Lindau (VHL) and other hereditary syndromes, active surveillance with delayed intervention is widely adopted, with nephron-sparing surgery typically triggered when the largest lesion reaches ~ 3 cm, a threshold associated with low metastatic risk under expert follow-up. In contrast, FH-deficient and high-grade SDH-deficient RCC are considered biologically aggressive, and current guidelines recommend immediate surgical excision rather than surveillance [35]. On the systemic-therapy side, prospective data now support bevacizumab plus erlotinib in FH-deficient RCC, with better objective responses and a median progression-free survival; notably, outcomes appeared more favorable in germline than in sporadic FH-deficient disease. Retrospective comparisons also suggest that immune checkpoint blockade can yield disease control rates at least comparable to VEGF-TKI monotherapy in FH-deficient RCC, providing a rationale for IO-based strategies in appropriate settings [36]. However, there are no dedicated, prospectively validated regimens for SDH-deficient RCC; treatment generally follows non-clear-cell RCC paradigms, and published experience remains limited to small retrospective series and case reports. Collectively, these points strengthen the clinical value of making a precise diagnosis, as it informs current treatment choices, enables referral to trials, and aligns oncologic care with germline risk management.

## Conclusions

This national survey, set against convergent international experience, shows a clear implementation gap: although SDH- and FH-deficient RCC are molecularly well defined and recognized in modern classifications, their translation into day-to-day diagnostics remains uneven across centers. Variability in access to dedicated IHC, heterogeneous interpretation standards, limited molecular confirmation, and inconsistent referral to clinical genetics collectively hamper timely and uniform recognition.

To close this gap, coordinated actions are needed on three fronts: (i) shared diagnostic guidelines between pathology, genetics, and oncology; (ii) educational initiatives to strengthen morphological literacy on metabolic RCC (e.g., curated digital slide repositories embedded within national and international digital pathology networks) to calibrate diagnostic thresholds and reduce inter-observer variability; (iii) detailed diagnostic algorithms to link histologic suspicion to ancillary testing and genetic counselling/cascade testing, ensuring that recognition reliably triggers appropriate hereditary cancer pathways.

Ultimately, the present survey provides a baseline against which future initiatives can measure progress in harmonizing practice, improving patient identification, and delivering integrated, risk-adapted care.

## Supplementary Information

Below is the link to the electronic supplementary material.

ESM 1 (PDF 5.51 MB)

## Data Availability

Original data are available from the corresponding author upon reasonable request.
