# Supplementary material for: Diagnostic practice and awareness of SDH- and FH-deficient renal cell carcinoma: results from an Italian Study Group of uropathology (GIUP) survey
Source: Virchows Arch. 2026 Feb 4;489(3):653–68. doi: 10.1007/s00428-026-04431-3 (PMC13582032; doi:10.1007/s00428-026-04431-3)
Supplement: Supplementary file 1 [file 428_2026_4431_MOESM1_ESM.pdf]

Diagnostic Practice and Awareness of SDH- and FH-Deficient Renal Cell Carcinoma: results from an Italian Study Group of Uropathology (GIUP) survey

Giuseppe Nicolò Fanelli[1,2,3]*, Anna Caliò[4]*, Stefano Marletta[5,6], Maria Ballotta[7], Marco Barella[8], Guido Bellezza[9], Paola Bianco[10], Angelo Giovanni Bonadio[11], Piergiuseppe Colombo[12,13], Alessandro D'Amuri[14,15], Giovanni De Chiara[16], Veronica Errigo[17], Denise Fiorini[18], Francesca Franzi[19], Daniele Liscia[20], Lisa Marcolini[18], Daniela Onnis[21], Francesca Pagliuca[22], Antonio Paniccià Bonifazi[23], Francesco Pierconti[24], Barbara Pozzi[25], Lavinia Stefanizzi[18], Marina Valeri[26], Mariavittoria Vescovo[27], Guido Martignoni[4,18#]

1. Division of Pathology, Department of Translational Research and New Technologies in Medicine and Surgery, University of Pisa, Pisa, Italy
2. First Division of Pathology, Pisa University Hospital, Pisa, Italy
3. Department of Pathology and Laboratory Medicine, Weill Cornell Medicine, New York, NY, USA
4. Department of Diagnostics and Public Health, Section of Pathology, University of Verona, Verona, Italy
5. Division of Pathology, Humanitas Istituto Clinico Catanese, Catania, Italy.
6. Department of Biomedical Sciences, Humanitas University, Milan, Italy.
7. Pathology Unit, AULSS 5 Polesana, S. Maria della Misericordia Hospital, Rovigo, Italy
8. Pathology Unit, Fondazione IRCCS Istituto Nazionale dei Tumori, Milan, Italy
9. Pathology Unit, Department of Medicine and Surgery, University of Perugia, Perugia, Italy
10. Pathology Unit, Department of Medical Sciences and Public Health, University of Cagliari, Cagliari, Italy
11. Pathology Unit, S. Giacomo Hospital, Novi Ligure, Italy
12. Department of Biomedical Sciences, Humanitas University, Milan, Italy.
13. Pathology Department, IRCCS Humanitas Research Hospital, Milan, Italy.
14. Pathology Unit, "Vito Fazzi" Hospital, Lecce, Italy
15. Pathology Unit, Department of Experimental Medicine, University of Salento, Lecce, Italy
16. Pathology Unit, A.O.R.N. "San Giuseppe Moscati", Avellino, Italy
17. Pathology Unit, Department of Pathology, San Paolo Hospital – ASL2 Liguria, Savona, Italy
18. Pathology Unit, Pederzoli Hospital, Peschiera del Garda, Italy
19. Pathology and Histology Unit, ASST Sette Laghi, Circolo and Fondazione Macchi Hospital, Varese, Italy
20. Pathology Unit, ASL Biella, Biella Hospital, Biella, Italy
21. Pathology Unit, ARNAS "G. Brotzu", Cagliari, Italy
22. Pathology Unit, University of Campania "Luigi Vanvitelli", Naples, Italy
23. Pathology Unit, ASL Roma 1, Rome, Italy
24. Institute of Pathology, Fondazione Policlinico Universitario A. Gemelli IRCCS and Catholic University of the Sacred Heart, Rome, Italy
25. Pathology Unit, ASST Lecco, Lecco, Italy
26. Pathology Unit, ASST Bergamo Est – Bolognini Hospital, Seriate, Bergamo, Italy
27. Pathology Unit, Fondazione Policlinico Universitario Campus Bio-Medico, Rome, Italy

* **Co-first author**

[#] **Corresponding author:** Guido Martignoni, Department of Diagnostics and Public Health, University of Verona, Largo L. Scuro 10, 37134 Verona, Italy. Email: guido.martignoni@univr.it

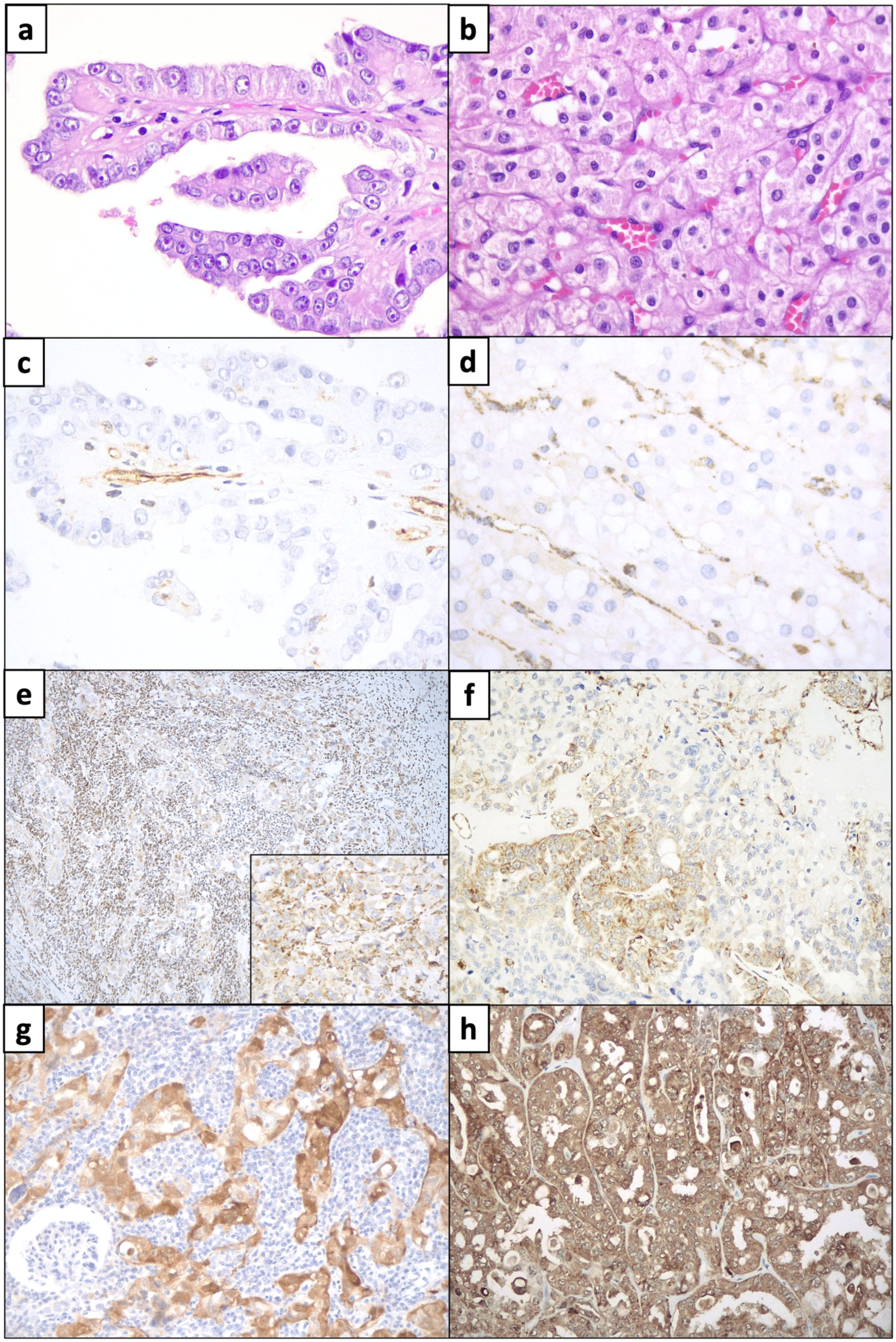
a
b
c
d
e
f
g
h

Supplementary Fig. 1 Morphology and immunohistochemistry in FH- and SDH-deficient RCC, including common pitfalls. (a) FH-deficient RCC, H&E, ×40. Eosinophilic cells with prominent macronucleoli and perinucleolar clearing within mixed/papillary architecture. (b) SDH-deficient RCC, H&E, ×40. Solid/nested oncocytic cells with architectural uniformity and finely vacuolated eosinophilic cytoplasm. (c) FH immunohistochemistry in FH-deficient RCC, ×40. Complete loss of FH in tumor cells with intact staining in internal positive controls (non-neoplastic tubules/endothelium). (d) SDHB immunohistochemistry in SDH-deficient RCC, ×40. Complete loss of SDHB in tumor cells with retained staining in vessels and non-neoplastic elements, confirming SDH-complex deficiency. (e) FH immunohistochemistry, "inflamed background" pitfall, ×10 (inset ×40). Nonspecific/background staining in inflamed or reactive stroma may mimic focal tumor positivity; interpretation must rely on internal controls. (f) FH immunohistochemistry, "focal/patchy tumor positivity" pitfall, ×20. Limited cytoplasmic labeling within otherwise negative tumor; such patterns can occur with pre-analytic variation or certain missense variants and should not override morphology or additional markers without appropriate controls. (g) 2-succinocysteine (2SC) immunohistochemistry in FH-deficient RCC, ×20. Strong, diffuse cytoplasmic and nuclear labeling supporting excess fumarate; inflamed areas may show variable background—interpret alongside FH loss and controls. (h) 2SC immunohistochemistry, "heterogeneous/focal labeling" pitfall, ×20. Suboptimal fixation or tissue heterogeneity can yield patchy 2SC reactivity; concordance with FH loss and internal controls is required. H&E, hematoxylin and eosin; IHC, immunohistochemistry; FH, fumarate hydratase; SDH(B), succinate dehydrogenase (B subunit); 2SC, 2-succinocysteine.
